# Hydroxyurea reduces the levels of the fetal *globin* gene repressors ZBTB7A/LRF and BCL11A in erythroid cells *in vitro*

**DOI:** 10.1093/jscdis/yoae008

**Published:** 2024-10-03

**Authors:** Gabriella E Martyn, Phillip A Doerfler, Yu Yao, Kate G R Quinlan, Mitchell J Weiss, Merlin Crossley

**Affiliations:** School of Biotechnology and Biomolecular Sciences, University of New South Wales (UNSW) Sydney, Sydney, NSW 2052, Australia; Department of Hematology, St Jude Children’s Research Hospital, Memphis, TN 38105-3678, United States; Department of Hematology, St Jude Children’s Research Hospital, Memphis, TN 38105-3678, United States; School of Biotechnology and Biomolecular Sciences, University of New South Wales (UNSW) Sydney, Sydney, NSW 2052, Australia; Department of Hematology, St Jude Children’s Research Hospital, Memphis, TN 38105-3678, United States; School of Biotechnology and Biomolecular Sciences, University of New South Wales (UNSW) Sydney, Sydney, NSW 2052, Australia

**Keywords:** SCD, hydroxyurea, fetal *globin*, BCL11A, ZBTB7A

## Abstract

**Objectives:**

Hydroxyurea (HU) is the most widely used therapy for adults and children with sickle cell disease (SCD). It is believed to act largely by inducing the transcription of fetal *γ-globin* genes to generate fetal hemoglobin (HbF), which inhibits the pathological polymerization of sickle hemoglobin (HbS). The mechanisms by which hydroxyurea elevates HbF are unclear. We explored the hypothesis that hydroxyurea induces HbF expression by inhibiting the expression of 2 *γ-globin* gene repressors, BCL11A and ZBTB7A (also known as LRF), which normally bind the *γ-globin* gene promoters to inhibit their expression after birth.

**Methods:**

We treated immortalized murine erythroleukemia cells and normal human donor CD34^+^ hematopoietic stem and progenitor cell-derived erythroblasts with hydroxyurea and measured the effects on globin, BCL11A and ZBTB7A protein and mRNA expression.

**Results:**

Treating murine erythroleukemia cells or human CD34^+^ hematopoietic stem and progenitor cell-derived erythroblasts with hydroxyurea reduced the protein levels of BCL11A and ZBTB7A compared to the vehicle-treated control. *BCL11A* mRNA levels were reduced in both cell types upon hydroxyurea treatment. However, *ZBTB7A* mRNA levels were only reduced in human CD34^+^ hematopoietic stem and progenitor cell-derived erythroblasts.

**Conclusions:**

Hydroxyurea can act in erythroid cells to reduce the levels and activity of two direct fetal *γ-globin* transcriptional repressors with accompanying de-repression of the *γ-globin* genes and induction of HbF, which may explain the mechanism of action leading to amelioration of symptoms in SCD patients treated with this drug.

## Lay Summary

Hydroxyurea (also known as hydroxycarbamide) is the most widely used treatment for children and adults with SCD. However, while it is known that treatment with hydroxyurea causes beneficial increases in fetal *globin* expression, it is not known how hydroxyurea leads to this effect. Two proteins are known to normally keep the expression of fetal *globin* silent in adult red blood cells. We showed here that hydroxyurea acts to reduce the amounts of these silencing proteins. Lower levels of these proteins could explain how hydroxyurea increases fetal *globin* expression in people with SCD and therefore the mechanism by which this drug works.

## Teaser

Hydroxyurea (also known as hydroxycarbamide) has been the most widely used treatment for children and adults with SCD since the 1980s. It is thought to act primarily by boosting the expression of fetal *globin*. The molecular mechanism through which hydroxyurea elevates fetal *globin* levels has not been understood, and we have explored this question here. It has recently been established that expression of the fetal *globin* gene is directly inhibited by 2 DNA-binding proteins, the transcriptional repressors ZBTB7A and BCL11A. We therefore tested—in primary human erythroid cells in culture and in cell lines—whether hydroxyurea affects the levels of these proteins. The results were clear. Both ZBTB7A and BCL11A protein levels were reduced by treatment with hydroxyurea. This important result is a first step toward elucidating the molecular mechanism through which hydroxyurea elevates fetal *globin* expression and an insight into why this small molecule with multiple potential activities can elevate fetal *globin* gene expression.

## INTRODUCTION

SCD, a devastating monogenic disorder affecting millions of individuals worldwide, is caused by a missense mutation in the *HBB* gene, which encodes the β-globin subunit of adult hemoglobin (HbA, α_2_β_2_).^1^^,^^2^ At low oxygen concentrations in venous capillaries, sickle hemoglobin (HbS, α_2_β^S^_2_) forms stiff polymers that cause red blood cells (RBCs) to assume a sickle shape and trigger a complex pathophysiology that includes hemolysis, vascular occlusion, and inflammation. Clinical consequences of SCD include progressive multi-organ damage, severe acute and chronic pain, and shortened lifespan.^2^

Hydroxyurea (HU) is a widely used as an effective medication for SCD. The drug inhibits ribonuclease diphosphate reductase, an enzyme that converts ribonucleotides to deoxyribonucleotides for DNA synthesis.^3^^,^^4^ Thus, HU inhibits cell proliferation by causing S-phase arrest and was initially used as a cancer treatment.^4^ However, it was demonstrated in 1984 that administration of HU to non-human primates^5^ or 2 patients with SCD^6^ resulted in de-repression of the fetal *γ-globin* genes (*HBG1* and *HBG2*) and increased levels of RBC fetal hemoglobin (HbF, α_2_γ_2_), which inhibits HbS polymerization.^7^ Subsequently, numerous clinical studies showed that HU therapy for SCD reduces pain crises, slows organ damage, lowers hospitalization rates, and prolongs survival.^8–10^ As a result of those studies, HU has been approved for the treatment of SCD in children and adults. Current National Heart, Lung and Blood Institute guidelines for SCD recommend that prophylactic HU therapy be administered to most patients over 9 months of age.^11^ While HU is thought to alleviate SCD pathophysiology mainly by inducing the expression of RBC HbF,^9^ other beneficial effects include reduction of blood cell adhesion,^12^ decreased neutrophil and platelet counts, and increased plasma nitric oxide levels.^2^

The mechanisms by which HU elevates HbF remain unclear. The drug does not reduce DNA methylation levels at the *β-globin* locus, which represents the proposed mechanism of HbF induction by 5-azacytidine.^5^^,^^6^ Another possibility is that HU-induced cell cycle arrest and cytotoxicity somehow program erythroid progenitors to express higher levels of *γ-globin* genes.^13^ Nitric oxide-related mechanisms for HbF induction by HU have also been proposed.^14^

Major insights into the developmental regulation of *globin* gene expression have occurred since HU was first used to treat SCD.^6^^,^^8^ In light of this information, we considered whether HU induces HbF expression by interfering with recently discovered mechanisms that regulate the perinatal *γ-globin* to *β-globin* switch. The tandem *γ-globin* genes *HBG2* (^G^γ) and *HBG1* (^A^γ) are highly expressed during fetal gestation and downregulated shortly after birth, with concomitant activation of the adjacent adult *β-globin* gene (*HBB*). This developmental switch is regulated by competition for a shared upstream enhancer, termed Locus Control Region.^15^ Accordingly, activation of *β-globin* automatically represses the *γ-globin* genes and vice versa. Two key transcriptional repressors, BCL11A^16^^,^^17^ and ZBTB7A,^18^ bind directly to cognate motifs in the *γ-globin* (*HBG*) gene promoters^19^^,^^20^ and recruit co-repressors, including the Nucleosome Remodeling and Deacetylase complex, to directly silence gene expression. Naturally occurring genetic variants in the binding sites for the BCL11A or ZBTB7A repressor proteins, located around positions –115 and –200 of the *γ-globin* promoter, respectively, cause sustained postnatal transcription, resulting in a benign genetic condition termed Hereditary Persistence of Fetal Hemoglobin (HPFH), which can alleviate the symptoms of co-inherited SCD.^19^

Recognition that genetic variants that disrupt BCL11A or ZBTB7A binding to the *γ-globin* (*HBG1* and *HBG2*) promoters cause HPFH indicates that these proteins are required for fetal *globin* gene repression. More recent studies have identified mechanisms that regulate the expression of *BCL11A*^21^^,^^22^ and *ZBTB7A*.^23^^,^^24^ We investigated whether HU treatment alters expression of *BCL11A* and *ZBTB7A* in immortalized murine erythroleukemia (MEL) cells and in human erythroblasts generated by *in vitro* differentiation of normal donor CD34^+^ hematopoietic stem and progenitor cell (HSPCs). Differences in *globin* gene switching exist between mice and humans. Specifically, mice undergo a single embryonic (*εy* and *βh1*)-to-adult (*β*^maj^ and *β*^min^) switch of *β-like globin* switch, whereas humans undergo embryonic (*ε-globin HBE*) to fetal (*γ-globin HBG1, HBG2*) to adult (*δ-* and *β-globin HBD, HBB*) *globin* switches.^25^ Importantly, BCL11A and ZBTB7A have a conserved role across species by serving as repressors of embryonic and fetal *globins*.^17^^,^^18^ In both MEL and CD34^+^ cells undergoing erythroid differentiation, treatment with HU caused reduced protein levels of BCL11A and ZBTB7A, accompanied by de-repression of mouse embryonic (ε*y*, β*h1*) or human fetal (*HBG1*, *HBG2*) *globin* genes. Thus, our results suggest that HU induces HbF in RBCs by reducing the levels of the 2 key fetal/embryonic *globin* gene repressors, BCL11A and ZBTB7A.

## MATERIALS AND METHODS

Experimental protocols are discussed briefly here and in greater detail in Supplementary Materials and Methods.

Murine erythroleukemia cells were treated with 100 μM HU for 48 h, then harvested for RNA extraction and qPCR analysis of *globin*, *Bcl11a*, and *Zbtb7a* transcript levels. Additionally, nuclear extracts were prepared and analyzed for BCL11A (NB600-261, Novus Biologicals) and ZBTB7A (sc-33683, Santa Cruz Biotechnology Inc.) protein levels via Western blot.

Normal human donor CD34^+^ HSPCs were treated with 0-40 μM HU on days 1, 3, or 8 of a 14-day protocol for erythroid differentiation. Cell surface markers for erythroid differentiation (CD235a^+^, CD49^+^, and Band3^+^) were assessed on days 8 and 14. Cells were harvested at day 7 for RNA extraction and qPCR analysis of *BCL11A*, *ZBTB7A*, *KLF1*, *GATA1*, and *globin* transcript levels. The primers used are provided in Supplementary Table S1. The *γ-globin* qPCR primers were designed to detect transcripts for both the *HBG1* and *HBG2* genes, which are highly related, whereas primers for adult *β-globin* detected only *HBB* mRNA. Additionally, CD34^+^ cells were harvested on day 10 for Western blot analysis of BCL11A (ab19487, abcam), ZBTB7A (sc-33683, Santa Cruz Biotechnology Inc), and GATA1 (ab11852, abcam) protein levels. CD34^+^ cells were harvested on day 14 to determine the percentage of HbF immunostaining cells (F-cells).

## RESULTS

**Figure 1.**
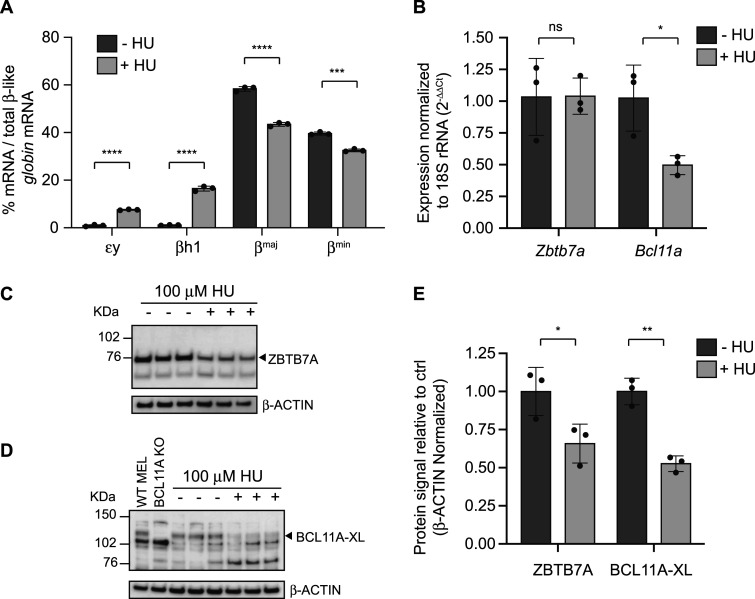
Hydroxyurea (HU) treatment of murine erythroleukemia (MEL) cells induces mouse embryonic/fetal β-like *globins* and reduces the levels of their transcriptional repressors ZBTB7A and BCL11A. (A) Mouse embryonic/fetal (ε*y* and β*h1*) and adult (β^*maj*^ and β^*min*^) β-like *globin* mRNA expression in MEL cells treated with HU. The mean ± SD is shown, with dots representing three experimental replicates for either vehicle-treated (–HU) or 100 μM hydroxyurea (+HU) treatment conditions. The *globin* percentages are shown as a proportion of the total β-like *globins* (sum of ε*y*, β*h1*, β^*maj*^, and β^*min*^). (B) Expression of *Zbtb7a* and *Bcl11a* mRNA in vehicle-treated (–HU) or hydroxyurea (+HU)-treated MEL cells. The mean ± SD is shown, with dots representing three experimental replicates. *Bcl11a* mRNA expression includes the XL, L, and S splice forms. (C and D) Western blot for ZBTB7A (C) and BCL11A-XL (D) proteins in vehicle-treated (–) or 100 μM HU (+)-treated MEL cells. Three independent experimental replicates are shown with β-ACTIN as a loading control. A MEL cell line where the entire BCL11A gene has been deleted via CRISPR-Cas9-mediated genome editing is included as a control and size standard (BCL11A KO). (E) Densitometry analysis for the ZBTB7A and BCL11A-XL Western blots shown in (C) and (D) with each symbol representing a separate experiment. Shown is the mean ± SD. Significance determined by an unpaired *t*-test is indicated as *P* > .05 (ns), *P* ≤ .05 (*), *P* ≤ .01 (**), *P* ≤ .0001 (****).

We first sought to verify previous studies showing that *in vitro* culture of adult-type mouse or human erythroid cells with HU causes de-repression of fetal *β-like globin* genes.^26^^,^^27^ Immortalized MEL cells express predominantly adult-type *β-like globin* genes, β^*maj*^ (*Hbb-b1*), and β^*min*^ (*Hbb-b2*). However, treatment with 100 μM HU for 48 h caused robust induction of the embryonic/fetal *globin* mRNAs ε*y* (0.93%-7.6%) and β*h1* (1.1%-16%), with a commensurate reduction in the adult β-like *globins* (Figure 1A). We next examined whether embryonic/fetal *globin* mRNA induction was associated with altered expression of *Zbtb7a* and/or *Bcl11a*, which repress human fetal *globin* genes (*HBG1* and *HBG2*) *in situ* and in transgenic mice, and mouse embryonic *globin* genes (ε*y* and β*h1*).^17^^,^^18^ BCL11A protein exists in multiple isoforms produced via alternative splicing, with the longest isoform (BCL11A-XL) being required for fetal *globin* gene repression.^16^ Treatment of MEL cells with HU caused no significant change in *Zbtb7a* mRNA compared to vehicle-treated cells (Figure 1B) and a 2-fold reduction in total *Bcl11a* mRNA levels (representing XL, L, and S isoforms). Western blot analysis of the same cells showed that ZBTB7A and BCL11A-XL proteins were both reduced by 34% and 47%, respectively, compared to the vehicle-treated condition (Figure 1C-E). These results demonstrate that HU treatment of an immortalized mouse erythroid cell line caused reductions in the mRNA levels of *Bcl11a* and protein levels of both BCL11A and ZBTB7A, which was accompanied by the de-repression of the mouse embryonic/fetal *globin* genes εy and β*h1*.

We next investigated the effect of HU on fetal *γ-globin* (*HBG1/2*), *ZBTB7A*, and *BCL11A* expression in human erythroid cells. First, we focused on immortalized human umbilical cord blood-derived erythroid progenitor-2 (HUDEP-2) cells,^28^ an adult-type erythroid cell line that expresses mainly β-*globin* (*HBB*). In contrast to findings in MEL cells, we did not observe any significant effect of HU treatment on the expression of *γ-globin* (*HBG1/2*), *ZBTB7A*, or *BCL11A* in 3 different clonally derived HUDEP-2 lines (Supplementary Figure S1). While the inability of HU to induce HbF in HUDEP-2 cells has been previously reported,^29^ the associated mechanism(s) are not known.

**Figure 2.**
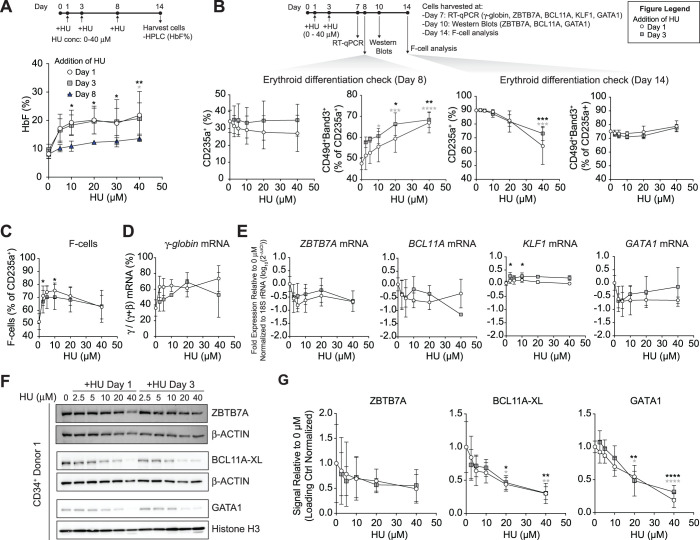
Treatment of human CD34^+^ cell-derived erythroblasts with hydroxyurea induces fetal *globin*s and reduces the levels of fetal *globin* repressors, ZBTB7A and BCL11A, and GATA1. Normal donor CD34^+^ hematopoietic stem and progenitor cells (HSPCs) (*n* = 3 independent donors) were induced to undergo erythroid differentiation on day 1. Hydroxyurea (HU) was added at the indicated concentrations (0-40 μM), beginning on either day 1, 3, or 8. (A) Fetal hemoglobin (HbF) protein levels determined by ion-exchange high-performance liquid chromatography (HPLC) on day 14. HbF is indicated as the proportion of fetal and adult hemoglobin (HbF/(HbF + HbA)). (B) HU dose-response measurements during the course of erythroid differentiation, with HU added on day 1 (open circle) or 3 (shaded square). Cells were analyzed for mRNA or protein in differentiation days 7 and 10, respectively, with erythroid differentiation assessed by flow cytometry on days 8 and 14 for maturation markers CD235a^+^ and CD49d^+^/Band3^+^ (as a percentage of CD235a^+^ cells). (C) F-cell levels were analyzed on day 14 by flow cytometry using an anti-HbF antibody. (D) *γ-globin* mRNA levels determined on day 7. The percentage of *γ-globin* (*HBG1/2*) is shown as a proportion of total β-like *globins* (γ /γ + β). (E) mRNA expression of *ZBTB7A*, *BCL11A* (XL, L, and S splice forms), *KLF1* and *GATA1* was determined on day 7. (F) Western blots for ZBTB7A (top), BCL11A-XL (middle), and GATA1 (bottom) determined on day 10. Days 1 and 3 indicate the day HU was added to the erythroid differentiation. Representative data from 1 CD34^+^ donor are shown. Supplementary Figure S2 shows the results of Western blots from 3 biological replicate experiments using 3 different CD34+ cell donors. β-ACTIN or Histone H3 are shown as loading controls. (G) Densitometry analysis for the ZBTB7A, BCL11A-XL, and GATA1 Western blots shown in (F). The mean ±SD are shown. Asterisks represent significance from a two-way ANOVA (relative to 0 μM HU) with Tukey’s multiple comparison testing. Black and grey asterisks represent significant differences between controls (0 μM) and HU added at either day 1 or day 3, respectively: (*) *P* ≤ .05, (**) *P* ≤ .01, (***) *P* ≤ .001, (****) *P* ≤ .0001.

Since HUDEP-2 cells did not respond to HU, we shifted to a different model and examined the effects of HU on *γ-globin* gene expression during *in vitro* erythroid differentiation of peripheral blood mobilized CD34^+^ HSPCs from three healthy donors. We induced the cells to undergo *in vitro* erythroid differentiation over 14 days, and different concentrations of HU (0-40 μM) were added on either day 1, 3, or 8. On day 14, we measured *γ-globin* mRNA, HbF protein by high-performance liquid chromatography, and HbF-immunostaining cells (F-cells), by flow cytometry. We observed a clear induction of HbF, with the addition of HU on either day 1 or 3, boosting HbF levels from 8.2% up to 23%. In contrast, adding HU on day 8 had minimal effect (Figure 2A). Next, we assessed the effects of different HU doses on erythroid differentiation and the expression of ZBTB7A, BCL11A, KLF1, and GATA1. KLF1 drives the expression of fetal *globin* repressors BCL11A and ZBTB7A, thereby silencing fetal *globin* expression and activating the expression of adult *β*-*globin.*^21–24^ GATA1 is a key erythroid transcription factor that stimulates BCL11A and ZBTB7A expression and is important for the developmental expression of *γ-globin*.^16^^,^^30–32^ The cell surface markers CD235a, Band3, and CD49d were used to monitor and compare erythroid differentiation of CD34^+^ cultures. On day 8, cultures treated with HU showed increased CD49d^+^/Band3^+^ erythroid cells despite little effect on CD235a expression, indicating accelerated erythroid differentiation (Figure 2B). However, erythroid cultures exposed to higher concentrations (20 or 40 μM) of HU added on day 1 or 3 showed fewer CD235a^+^ cells on day 14, indicating abnormal or arrested erythroid cell development.

The proportion of F-cells and HbF levels were relatively high at baseline, as previously reported for erythroblasts derived by *in vitro* differentiation of CD34^+^ cells.^33^ We observed the strongest induction of HbF on day 14 of erythroid differentiation with the addition of 10 μM HU on day 1. Under these conditions, the proportion of F-cells increased from 52% to 75% and fetal *γ-globin (HBG1/2)* mRNA increased 1.8-fold compared to vehicle-treated control cells (Figure 2C and D). At this concentration of HU, the degree of erythroid maturation was similar to that of control cells (Figure 2B). The more modest and variable F-cell induction at higher HU concentrations may reflect cytotoxicity or the reduced proportion of CD235a^+^ erythroid cells present. At the same time, 10 μM HU added on day 1 caused a 62% reduction in *ZBTB7A* mRNA and a 61% reduction in *BCL11A* mRNA (XL, L, and S isoforms) compared to vehicle-treated cells (Figure 2E). Similar to what we observed in mouse MEL cells, HU treatment caused reductions in the protein levels of ZBTB7A and BCL11A, by 31% and 39%, respectively, as determined by Western blot analysis (Figure 2F and G, Supplementary Figure S2).

KLF1 indirectly represses HbF by stimulating the expression of *γ-globin* repressor genes *BCL11A* and *ZBTB7A*.^21^^,^^24^ Suitable antibodies to assess KLF1 protein levels by Western blotting or chromatin immunoprecipitation were unavailable. However, we observed an 11% increase in KLF1 mRNA levels when 10 μM HU was added on day 1 compared to vehicle-treated control cells (Figure 2E). This finding indicates that HU does not induce HbF by suppressing *KLF1* gene transcription. While it is possible that HU suppresses KLF1 expression post-transcriptionally, it is more likely that HU-associated reductions in BCL11A and ZBTB7A expression occur through KLF1-independent effects.

We also considered the effects of HU on GATA1, a key erythroid transcription factor that drives *BCL11A* and *γ-globin* expression during development.^16^^,^^30^^,^^31^^,^^34^ GATA1 levels decreased after HU treatment (by 66% at the RNA level and by 30% at the protein level). Taken together, these results show that HU treatment of CD34^+^ cell-derived erythroblasts causes de-repression of fetal *HBG1/2 γ-globin* transcription and elevated HbF, which are associated with reductions in *ZBTB7A* and *BCL11A* mRNA and protein, increased *KLF1* mRNA level, and reduced *GATA1* mRNA. Furthermore, our results show that concentrations of HU above 10 μM inhibit *in vitro* erythroid differentiation of CD34^+^ HSPCs.

## DISCUSSION

HU is the most widely used pharmacologic therapy for SCD. The drug is well-tolerated and thought to exert its therapeutic effect primarily by elevating fetal *globin* and HbF levels, which ameliorates SCD pathophysiology and symptoms by interfering with HbS polymerization.^6–10^ However, the drug also exerts complex and widespread effects on hematopoiesis. By inhibiting DNA replication and causing cell cycle arrest, HU has been proposed to trigger a form of “stress erythropoiesis” via unidentified signaling pathways that somehow activate the expression of *γ-globin (HBG1/2)* and HbF during adult erythropoiesis.^35^^,^^36^ Our results showed that high concentrations of HU led to aberrant *in vitro* differentiation of erythroid precursors derived from primary human CD34^+^ HSPCs. Overall, the precise molecular pathways through which HU increases fetal *globin* expression and HbF production have remained elusive.

Here, we show that in erythroid cells, HU causes a reduction in both major fetal *globin* gene repressors: BCL11A and ZBTB7A. It is now well-established that full expression of BCL11A (XL isoform) is required for fetal *globin* gene repression.^16^ Common single-nucleotide polymorphisms in an erythroid-specific enhancer within intron 2 of the *BCL11A* gene are associated with increased HbF expression in normal adult RBCs.^37^ Extensive evidence from erythroblasts grown *in vitro*, mouse models, and human genetic studies show that HbF levels are exquisitely responsive to BCL11A levels.^16^^,^^17^^,^^38–40^ For example, *BCL11A* haploinsufficiency in humans is associated with elevated HbF levels.^41^^,^^42^ *ZBTB7A* is also required for full fetal *globin* gene repression, and loss of greater than 50% ZBTB7A protein levels has a significant impact on fetal *globin* gene repression in mice harboring a yeast artificial chromosome containing the entire extended β-like *globin* locus and in human CD34^+^ cell-derived erythroid progenitors.^18^ Our findings that HU treatment causes a reduction in BCL11A and ZBTB7A proteins fit with the observations that HbF is significantly elevated in erythroid cells after HU treatment.

Our findings are consistent with other studies indicating that HU can induce HbF by reducing BCL11A expression levels. Zhang *et al.*^27^ observed reductions in BCL11A in CD34^+^ cells after HU treatment. The same study showed reduced CD235a^+^ expression in erythroid cultures treated with 30 μM HU. In a clinical study, *BCL11A* mRNA levels were reduced in young reticulocytes from children with SCD who were receiving maximum tolerated doses of HU.^43^ Furthermore, other studies indicate that single-nucleotide polymorphisms in the *BCL11A* intron 2 erythroid enhancer are associated with the magnitude of HbF induction during HU therapy for SCD.^44^^,^^45^ Another study showed that HU treatment of basophilic erythroblasts derived from bone marrow common myeloid progenitors resulted in the induction of *γ-globin* mRNA and reductions in the levels of *BCL11A* and erythroid transcription factors *KLF1* and *TAL1*, with no significant differences in the levels of *GATA1*, CD71 (transferrin receptor), or CD235a (glycophorin A).^46^ This contrasts with our study using mobilized CD34^+^ HSPC-derived erythroid progenitors in which we observed a reduction in CD235a^+^ erythroid cells, increased *KLF1* mRNA levels, and reduced GATA1 mRNA and protein levels. Perhaps the dose of HU might explain some of the discrepancies between our observations and this previously reported study.^46^ With both KLF1 and TAL1 having established roles in hemoglobin switching and erythropoiesis more broadly,^21^^,^^47^ it is likely that HU treatment alters multiple transcription factors to modulate *γ-globin* (*HBG1/2*) expression. It is also possible that the reductions in GATA1 levels observed in our experiments (Figure 2G) contribute to the decrease of *BCL11A* mRNA in MEL cells and primary erythroblasts, as GATA1 is known to be a key driver of BCL11A.^31^^,^^37^ Our findings are consistent with observations that BCL11A expression is sensitive to GATA1 levels. For instance, single-nucleotide variants that reduce GATA1 binding to a BCL11A enhancer reduce BCL11A levels enough to raise fetal *globin* expression.^37^ But the impacts on ZBTB7A and KLF1 appear to be more complicated, including a post-translational reduction in the case of ZBTB7A in MEL cells and a modest increase in *KLF1* mRNA in human CD34^+^ cell-derived erythroblasts. Accordingly, the situation is complex and the precise mechanisms by which HU reduces GATA1 levels and affects its target genes to different extents requires further investigation. It will also be interesting to study the effects of HU on the expression of KLF1 protein, which is known to stimulate BCL11A and ZBTB7A production.^21^^,^^24^

Our study has several limitations. First, the benefits of HU, including induction of HbF, appear to be greater in SCD patients than the effects on cell lines. This may be due to differences in drug exposure, including *in vitro* toxicities on cells exposed to high concentrations of HU continuously. Moreover, different erythroid cell lines responded differently to HU in our study. We are unable to explain why HU fails to induce HbF in human HUDEP-2 cells or observed differences in the regulation of ZBTB7A expression by HU in CD34^+^ cell-derived erythroblasts vs MEL cells. Second, CD34^+^ cell-derived erythroblasts exhibit relatively high baseline levels of HbF and F-cells,^33^ which vary according to different donors and can obscure the effects of HU. Third, we did not examine the effects of HU on erythroblasts generated from SCD donor CD34^+^ cells. Compared to healthy donor erythroblasts, those from individuals with SCD exhibit higher baseline levels of HbF that may have additive or synergistic effects with HU. Despite these limitations, our findings that HU reduces the levels of the key fetal *globin* gene repressors ZBTB7A/LRF and BCL11A likely provide insight into the associated mechanism for HbF induction *in vivo.*

## CONCLUSION

Our data demonstrate in controlled *in vitro* systems that HU treatment reduces the levels of the key fetal *globin* gene repressors BCL11A and ZBTB7A. While other models have been previously proposed, such as the induction of “stress erythropoiesis” model, our new findings suggest that altered levels of key transcription factors that regulate β-like *globin* gene expression may be a major erythroid cell intrinsic mechanism by which HU reactivates fetal *globin* gene expression and elevates HbF expression in adult-type erythroid cells.

## Supplementary Material

yoae008_Supplementary_Data

## Data Availability

Requests for data sharing may be submitted to Merlin Crossley (m.crossley@unsw.edu.au) or Mitchell Weiss (weissmi@stjude.org).
