# Supplementary material for: Hydroxyurea reduces the levels of the fetal globin gene repressors ZBTB7A/LRF and BCL11A in erythroid cells in vitro
Source: J Sick Cell Dis. 2024 Oct 3;1(1):yoae008. doi: 10.1093/jscdis/yoae008 (PMC12039817; doi:10.1093/jscdis/yoae008)
Supplement: yoae008_Supplementary_Data [file yoae008_Supplementary_Data.docx]

**Supplementary Materials**

**Manuscript Title:** Hydroxyurea reduces the levels of the fetal *globin* gene repressors ZBTB7A/LRF and BCL11A in erythroid cells *in vitro*

**Authors and affiliations:** Gabriella E. Martyn PhD^#^1^, Phillip A. Doerfler PhD^#2^, Yu Yao MD, PhD^2^, Kate G.R. Quinlan PhD^1^, Mitchell J. Weiss MD, PhD^2*^, Merlin Crossley DPhil^1*^

^#^GEM and PAD contributed equally as co-first authors

*MJW (mitch.weiss@stjude.org) and MC (m.crossley@unsw.edu.au) contributed equally as corresponding authors

^ Now at *BASE Initiative, Betty Irene Moore Children's Heart Center, Lucile Packard Children's Hospital, Stanford University School of Medicine, Stanford, CA, USA and Department of Genetics, Stanford University School of Medicine, Stanford, CA, USA.*

1. *School of Biotechnology and Biomolecular Sciences, University of New South Wales (UNSW) Sydney, Sydney, New South Wales, 2052, Australia.*
2. *Department of Hematology, St. Jude Children’s Research Hospital, Memphis, TN, 38105-3678, USA*

**Corresponding author mailing address and email:** Merlin Crossley, School of Biotechnology and Biomolecular Sciences, Room 3105, Level 3, Biosciences South Building (E26), UNSW Sydney, NSW, 2052, Australia. m.crossley@unsw.edu.au

**Cell culture and Hydroxyurea (HU) treatment**

**MEL cells and HU treatment:** Murine erythroleukemia (MEL) cells were cultured in RPMI1640 media (*Invitrogen-Life Technologies*), supplemented with 10% fetal calf serum (FCS, *Gibco-Life Technologies*) and 1% penicillin, streptomycin and glutamine (PSG, *Gibco-Life Technologies*). Cells were incubated at 37°C in a 5% CO_2_ water jacketed incubator while culturing.

MEL cells were treated with a final concentration of 100 μM of hydroxyurea (HU) for 48 hours before subsequent experiments were performed. Briefly, 5 x 10^6^ cells were seeded per 15 cm tissue culture dish containing 25 mL of complete RPMI640 media (10% FCS, 1% PSG). Hydroxyurea was dissolved in Milli-Q water to make a working stock solution. The +HU condition received the hydroxyurea drug (100 μM) while the ‑HU condition were ‘mock’ treated with the same volume of Milli-Q water. Three biological replicates were set up for each condition (- HU / + HU). A total of 20 million cells were treated for the -HU condition and 30 million cells for the +HU condition. After 48 hours of treatment, cells were harvested for the various experiments: RNA extraction, cDNA synthesis and qPCR and Western Blots.

**HUDEP-2 and HU treatment:** Human Umbilical Cord Derived Erythroid Progenitor 2 (HUDEP-2) cells were grown in StemSpan^TM^ Serum Free Expansion Media (SFEM) (*Stemcell Technologies*) supplemented with SCF (50 ng/ml) (*R&D Systems*), EPO (3 IU/ml) (*Peprotech*), dexamethasone (10^−6^ M) and doxycycline (1 µg/ml). Medium was made up fresh every time before use and supplemented with 1% penicillin, streptomycin and L-glutamine (PSG). Cells were maintained at a concentration of 5 × 10^5^ cells/ml.

Approximately, 2x10^6^ HUDEP-2 cells were seeded per 10 cm tissue culture dish and were treated with a final concentration of 100 μM of hydroxyurea (HU) for 48 hours. We have observed that populations of ‘WT’ HUDEP-2 cells can express various levels of γ-*globin* at a mRNA level and therefore decided to start with three ‘WT’ HUDEP-2 clonal populations which expressed low levels of γ-*globin* (~0.4-1.2%). The HU drug treatment was applied to these three HUDEP-2 WT clonal populations (+HU condition) or the same volume of Milli-Q water was added as a control to the mock treated cells (-HU condition).

**CD34^+^ cells and differentiation:**

Mobilized peripheral blood CD34^+^ cells were obtained from de-identified healthy donors (*StemExpress*, LLC, Folsom, CA) and enriched by immunomagnetic selection using an AutoMACS instrument (*Miltenyi Biotec*).

Erythroid differentiation was induced using a two-phase system. Cryopreserved CD34^+^ cells were thawed into phase 1 medium. Phase 1 (days 0-7) was carried out using IMDM (*ThermoFisher*) supplemented with 2% human AB plasma (SeraCare, 1810-0001), 3% human AB serum ((Atlanta Biologicals, S40110), 3 U/mL heparin (Sagent Pharmaceuticals, NDC 25021-401-02), 200 μg/mL holo-transferrin (Millipore Sigma, T0665), 3 U/mL EPO (Amgen, EPOGEN NDC 55513-144-01), 10 ng/mL SCF (R&D systems, 255-SC/CF), and 1 ng/mL IL-3 (R&D systems, 203-IL/CF). For phase 2 (days 8-14) IL-3 is omitted from the medium. Hydroxyurea (H8627, Millipore-Sigma) was prepared fresh in water for each experiment and CD34+ cells were treated with 0-40 μM of HU on days 1, 3 or 8 of a 14 day protocol for erythroid differentiation. Cultures were maintained at 0.5-1x10^6^ cells/mL for phase 1 and 1-2x10^6^ cells/mL for phase 2.

*In vitro* erythroid differentiation of CD34^+^ cells was measured by flow cytometry for the cell surface markers CD235a (ThermoFisher, 11-9886-42), CD49d (BioLegend, 304322), and Band3 (a gift from X. An, New York Blood Center). For F-cell analysis, cells were fixed with 0.05% glutaraldehyde (Millipore Sigma, catalog no. G5882), and permeabilized with 0.1% Triton X-100 (Millipore Sigma, catalog no. 93443). Subsequently, cells were stained with antibodies against human CD235a and HbF (Invitrogen, MHFH05). Data were collected using BD FACS Diva software on a BD Symphony A3 and analysed using FlowJo.

**COS-7:** COS-7 cells were cultured in DMEM (*Gibco-Life Technologies*) supplemented with 10% FCS and 1% penicillin-streptomycin-glutamine (PSG). As the cells are adherent, they were dislodged after a 5-minute incubation at 37 °C with 0.05% trypsin-EDTA (*Invitrogen-Life Technology*). Plasmids were transiently transfected into COS-7 cells to over-express BCL11A and ZBTB7A proteins as size marker controls for Western blots (see transfection of COS-7 cells for more details).

**Transfection of COS-7 cells:**

Transcription factors BCL11A and ZBTB7A were transiently overexpressed in COS-7 cells, and nuclear extracts were prepared (see Preparation of Nuclear Extracts and Western Blots for more details). 10 cm plates of COS-7 cells were transfected with 5 μg of vector using Fugene 6 (*Promega*). The mammalian expression vectors used were: pcMV6-Entry Myc-DDK-tagged ORF Homo Sapiens BCL11A (RC219567, *Origene*) and pcMV6-XL6-human ZBTB7A (sc-114581, *Origene*). The empty vector pcDNA3 was transfected as a control. At 48 hours after transfection, nuclear extracts were harvested.

**RNA extraction, cDNA synthesis and quantitative PCR (qPCR)**

**Details for MEL and HUDEP-2 cells:** Cells were pelleted and washed with PBS before total RNA was extracted. Total RNA was extracted from cells with TRI-Reagent (*Sigma Aldrich Company*) and by using the RNeasy mini kit (*Qiagen*) and the Rnase-free Dnase kit (*Qiagen*), as per the manufacturer’s instructions. Approximately 1 μg of RNA for MEL samples, or 500 ng of RNA for HUDEP-2 samples was used for complementary DNA (cDNA) synthesis using Super-Script^TM^ VILO^TM^ Master Mix kit (*ThermoFisher*). A sample without the Superscript III reverse transcriptase enzyme was also set up for each sample as a negative control, to detect the presence of genomic DNA contamination. cDNA was diluted 1:400 before qPCR was performed using Power SYBR Green PCR Master Mix. The reaction was run on the ViiA 7 Real-Time PCR System (*ThermoFisher Scientific*) using default cycle parameters. The qPCR primers used to determine the expression levels of the *globin* genes are listed in Table S1 below.

**Details for CD34+ cells:** For qRT-PCR studies, 0.5x10^6^ cells were collected from day 7 cultures of CD34^+^ cells. Total RNA was extracted using a Rneasy Plus Mini Kit (*Qiagen*). For cDNA synthesis, 200 ng of RNA was used for reverse transcription using the iScript cDNA synthesis kit (*Bio-Rad*) following the manufacturer’s instructions. The cDNA was diluted to 2 ng/μL for qRT-PCR using primers for *ZBTB7A*, *BCL11A, KLF1, GATA1*, *HBB*, *HBG1/2*, and *18S rRNA* (Table S1).

**Analysis of qPCR data:** The expression levels of the genes of interest were normalized against *18S rRNA* levels. The fold change in gene expression levels was calculated using the ΔΔC_T_ method. Percent *HBG1/2* was calculated using the relative proportion of *HBB* and *HBG1/2* following fold change (ΔC_T_) determination relative to *18S rRNA*.

**Table S1.** qPCR primers used to determine the levels of the β-like *globin* genes and transcription factors BCL11A, ZBTB7A, KLF1 and GATA1. Sequences for the forward (F) and reverse (R) primers are shown and the species is also indicated.

| **Gene** | **Number** | **F/R** | **Sequence** |
| --- | --- | --- | --- |
| 18S rRNA | A1560 | F | CACGGCCGGTACAGTGAAAC |
|  | A1561 | R | AGAGGAGCGAGCGACCAA |
| Hbb-b1  (mouse) | B1250 | F | GGAAAGGTGAACTCCGATGAA |
|  | B1251 | R | TGATAGCAGAGGCAGAGGATAG |
| Hbb-b2  (mouse) | A3395 | F | GCACCTGACTGATGCTGAGA |
|  | A3396 | R | CTGGGTCCAAGGGTAGACAA |
| Hbb-βh1  (mouse) | B1260 | F | GGAGAAGGCAGCTATCACAA |
|  | B1261 | R | GAATCTCTGAGTCCATGGGTAAA |
| Hbb-y  (mouse) | B1256 | F | CCTTGGGAAGGCTTCTTGTT |
|  | B1257 | R | CCCTTGGGTTGCCCATTAT |
| γ-*globin*  (human) | A2524 | F | CCTGTCCTCTGCCTCTGCC |
|  | A2525 | R | GGATTGCCAAAACGGTCAC |
| β-*globin*  (human) | B1228 | F | CACGTGGATCCTGAGAACTT |
|  | B1229 | R | CCAGCCACCACTTTCTGATA |
| mBCL11A  (mouse) | A3431 | F | CCAGAGGATGACGATTGTTTATCA |
|  | A3432 | R | GAAGTTTATCTGCTATGTGTTCCTGTTT |
| mZBTB7A  (mouse) | A5039 | F | GCGACGTGGTGATTCTTGTG |
|  | A5040 | R | CGACGTGAACAGCTTCTTGAAGT |
| hBCL11A  (human) | A4111 | F | CGAGCACAAACGGAAACAATG |
|  | A4112 | R | GATTAGAGCTCCATGTGCAGAACG |
| hZBTB7A  (human) | A5041 | F | AAGCCCTACGAGTGCAACATCT |
|  | A5042 | R | CAGGTCGTAGTTGTGGGCAAA |
| hKLF1  (human) |  | F | GGTGTGATAGCCGAGAC |
|  |  | R | GCGTATGGCTTCTCCC |
| hGATA1  (human) |  | F | CTGTCCCCAATAGTGCTTATGG |
|  |  | R | GAATAGGCTGCTGAATTGAGGG |

**Preparation of Nuclear Extracts and Western Blots for MEL and HUDEP-2 cells**

Western blots were performed as previously described [(*1*)](https://paperpile.com/c/80ry0s/TyqXS). Briefly, nuclear extracts were prepared and 20-40 μg of protein (or 5-10 μg of protein for nuclear extracts from the over-expression of proteins in COS-7 cells) was boiled with 1 x NuPAGE LDS Sample Buffer (*ThermoFisher*) (supplemented with a final concentration of 0.1M DTT) at 95°C for 5 minutes. Samples were loaded onto a 4-12% NuPAGE^®^ Bis-Tris Precast Gel (*ThermoFisher*) and run in NuPAGE^®^ MES SDS Running Buffer (*ThermoFisher*) at 200 volts for at least 90 minutes for BCL11A blots or 60 minutes for ZBTB7A blots. Rainbow^TM^ protein standards were loaded on each gel for size estimation. A control MEL cell line where the entire BCL11A gene has been deleted via CRISPR-Cas9 mediated genome editing (MEL BCL11A KO) was also used as a control and size standard. Proteins were transferred onto 0.2 μM PVDF (activated in methanol) blotting membrane using transfer buffer (25 mM Tris, 0.2 M glycine and 20% (v/v) methanol) at 30 volts for 60 minutes. The PVDF membrane was blocked with 3% (w/v) skim milk in TBST (50 mM Tris-HCl (pH 7.4), 150 mM NaCl and 0.05% Tween 20) for 2 x 15 minutes, followed by 2 x 15 minute washes in TBST. Next the membrane was incubated with the primary antibody, as outlined in Table S2. 5 x 5 minute washes were performed on the membranes with TBST prior to a 1-2 hour incubation with the appropriate secondary horseradish peroxidase-linked antibody for the species, diluted in TBST (See Table S2). 5 x 5 minute washes were conducted again on the membranes before detection The Immobilon^TM^ Western Chemiluminescent HRP substrate kit was used for Western blot visualization on the ImageQuant^TM^ LAS 500 imager (*GE Healthcare Life Sciences*). Blots were stripped with 0.2 M NaOH for 10 minutes, before blocking again with skim milk re-probing for β-ACTIN. Protein quantification was normalized to ACTIN and represented as a proportion of signal relative to vehicle-treated cultures using Image Lab (*v6.1.0, Bio-Rad*).

**Table S2.** Primary and secondary antibodies used for the BCL11A and ZBTB7A Western Blots performed in MEL and HUDEP-2 cells, treated with HU. The dilutions and incubation times are also specified.

| **Antibody** | **Target** | **Dilution** | **Incubation Time** | **Manufacturer, Product No.** |
| --- | --- | --- | --- | --- |
| Primary Antibody | BCL11A | 1:5,000 in TBST | 2 hours | NB600-261, *Novus Biologicals* |
|  | ZBTB7A | 1:12,500 in TBST | 1 hour | sc-33683, *Santa Cruz Biotechnology Inc.* |
|  | β-ACTIN | 1:30,000 in TBST | 1 hour | A1978, *Sigma Aldrich Company* |
| Secondary Antibody | ECL^TM^ Anti-rabbit IgG HRP | 1:15,000 in TBST | 1 hour | Cat # NA934D  *GE Healthcare Life Sciences* |
|  | HRP-Anti-Armenian hamster | 1:5,000 in TBST | 1 hour | sc-2443, *Santa Cruz Biotechnology* |
|  | ECL^TM^ Anti-mouse IgG HRP | 1:10,000 in TBST | 1 hour | Cat # NA931V  *GE Healthcare Life Sciences* |

**High Performance Liquid Chromatography for CD34^+^ cells**

For ion-exchange high-performance liquid chromatography, 1x10^6^ cells were collected from day 14 cultures of CD34^+^ cells. Cell lysates were prepared using hemolysate reagent (*Helena Laboratories*) and analyzed using ion-exchange columns on a Prominence HPLC system (*Shimadzu*). Proteins eluted from the column were identified at 220 and 415 nm with a diode array detector. The relative amounts were calculated from the area under the 415nm peak and normalized based on the dimethylsulfoxide control. Data was collected and analyzed using LabSolutions Software (*v.5.81 SP1, Shimadzu*). The percentage of fetal hemoglobin was calculated as a proportion of both fetal and adult hemoglobin.

**Protein extraction and Western Blots for CD34^+^ cells**

For protein extraction, 5x10^6^ cells were collected from day 10 cultures. Cells were washed in PBS and resuspended in 10× packed cell volume of complete hypotonic lysis buffer (10 mM HEPES, pH 7.9, 1.5 mM MgCl_2_, 10 mM KCl, 5 mM dithiothreitol, 1 mM phenylmethylsulfonyl fluoride, 1X protease inhibitor cocktail [P8340*; Sigma*]). The cells were incubated on ice for 10 minutes, thoroughly vortexed, pelleted at 420 x *g* for 5 minutes, and the supernatant discarded. The pellet was resuspended in 2× to 3× packed cell volume of complete extraction buffer (20 mM HEPES, pH 7.9, 1.5 mM MgCl_2_, 0.42 M NaCl, 0.2 mM EDTA, 25% glycerol with 5 mM dithiothreitol, 1 mM phenylmethylsulfonyl fluoride, 1X protease inhibitor cocktail) and incubated on ice for 20–30 minutes. The suspension was then centrifuged at 16,000 x *g* for 5 minutes at 4°C and the supernatant recovered.

Extracts were subject to SDS-PAGE using 4-12% Bis-Tris polyacrylamide gels (*ThermoFisher*). For Western blot analysis, proteins were transferred to PVDF membranes, blocked in 3% milk+TBS-Tween20 for 1 hour at room temperature, with overnight primary antibody incubation at 4°C with shaking. Following primary incubation, blots were washed 3 x 5 minutes using TBS-Tween20 at room temperature. Secondary antibody incubation was performed for 2 hours at room temperature with shaking. Blots were incubated with Immobilon Crescendo Western HRP substrate (*Millipore*) using the manufacturer’s suggestions. Blots were imaged using chemiluminescence with a ChemiDoc Imaging System (*Bio-Rad*). Protein quantification was normalized to ACTIN or histone H3 and represented as a proportion of signal relative to 0 μM HU treated cultures using Image Lab (v6.1.0, Bio-Rad). Antibodies were diluted in 3% milk+TBS-Tween20. The antibodies and dilutions used for the CD34+ Western Blots are highlighted in Table S3.

**Table S3.** The antibodies used for the BCL11A and ZBTB7A Western Blots performed in CD34^+^ cells, treated with HU.

| **Target** | **Dilution** | **Manufacturer, product no.** |
| --- | --- | --- |
| ACTIN | 1:5000 | Millipore-Sigma, A2228 |
| Histone H3 | 1:20000 | abcam, ab1791 |
| ZBTB7A / LRF | 1:1000 | Santa Cruz, sc-33683 |
| BCL11A | 1:1000 | abcam, ab19487 |
| GATA1 | 1:1000 | abcam, ab11852 |
| Goat anti-Mouse-HRP | 1:10000 | Thermo, A28177 |
| Goat anti-Rabbit-HRP | 1:10000 | Thermo, A27036 |
| Goat anti-Armenian Hamster HRP | 1:10000 | Novus Biologicals, NB100 |

**References**

1. [J. Sambrook, E. F. Fritsch, T. Maniatis, *Molecular Cloning: A Laboratory Manual* (Cold Spring Harbor Laboratory, 1989).](http://paperpile.com/b/80ry0s/TyqXS)

**Supplementary Figure 1. Hydroxyurea does not induce γ-*globin* expression or alter ZBTB7A and BCL11A protein levels in human HUDEP-2 erythroid cells.** (A) γ-*globin* (*HBG1/2*) mRNA levels in vehicle treated (-HU) and 100 μM hydroxyurea treated (+HU) clonally derived HUDEP-2 cell-lines (clonal cell lines 3, 4 and 5). Shown is the percentage of γ-*globin* as a proportion of total β-like *globins* (γ (*HBG1/2)* + β (*HBB)*). (B) Western blots for ZBTB7A and BCL11A-XL in vehicle treated (-) or 100 μM HU (+) treated HUDEP-2 cells. Nuclear extracts from COS cells over-expressing BCL11A-XL with a C-terminal FLAG-tag (BCL11A-FLAG) and ZBTB7A construct are included as size controls. β-ACTIN is shown as a loading control. (C) Densitometry analysis for the ZBTB7A and BCL11A-XL Western blots shown in (B). Shown is the mean $\pm$S.E.M with dots representing data from three independent HUDEP-2 clonal cell-lines (n=3).

**Supplementary Figure 2. Hydroxyurea treatment of human CD34^+^ cell-derived erythroblasts reduce ZBTB7A, BCL11A and GATA1 protein levels.** Western blots for ZBTB7A (A), BCL11A-XL (B) and GATA1 (C) are shown for vehicle treated (0 μM) and 2.5 μM – 40 μM hydroxyurea (HU) treated CD34^+^ cell-derived erythroblasts. HU has either been added on day 1 or day 3 of the erythroid differentiation. β-ACTIN or Histone H3 is shown as a loading control. Shown is the complete set of data from three independent CD34^+^ donors (related to Figure 2F and 2G).
